# Extracts of Amazonian Fungi With Larvicidal Activities Against *Aedes aegypti*

**DOI:** 10.3389/fmicb.2021.743246

**Published:** 2021-12-10

**Authors:** Marta Rodrigues de Oliveira, Ricardo de Melo Katak, Gilvan Ferreira da Silva, Osvaldo Marinotti, Olle Terenius, Wanderli Pedro Tadei, Afonso Duarte Leão de Souza, Antonia Queiroz Lima de Souza

**Affiliations:** ^1^Programa de Pós-graduação em Biodiversidade e Biotecnologia (PPG-BIONORTE), Universidade Federal do Amazonas, Manaus, Brazil; ^2^Programa de Pós-graduação em Biotecnologia, Universidade Federal do Amazonas, Manaus, Brazil; ^3^Embrapa Amazônia Ocidental, Manaus, Brazil; ^4^MTEKPrime, Aliso Viejo, CA, United States; ^5^Department of Cell and Molecular Biology, Microbiology, Uppsala University, Uppsala, Sweden; ^6^Laboratório de Malária e Dengue, Instituto Nacional de Pesquisas da Amazônia, Manaus, Brazil; ^7^Central Analítica – Centro de Apoio Multidisciplinar, Universidade Federal do Amazonas, Manaus, Brazil; ^8^Departamento de Química, Universidade Federal do Amazonas, Manaus, Brazil; ^9^Faculdade de Ciências Agrárias, Universidade Federal do Amazonas, Manaus, Brazil

**Keywords:** biological control, arbovirus, *Aedes aegypti*, larvicidal activity, metabolites

## Abstract

The global increase in diseases transmitted by the vector *Aedes aegypti*, new and re-emerging, underscores the need for alternative and more effective methods of controlling mosquitoes. Our aim was to identify fungal strains from the Amazon rain forest that produce metabolites with larvicidal activity against *Aedes aegypti*. Thirty-six fungal strains belonging to 23 different genera of fungi, isolated from water samples collected in the state of Amazonas, Brazil were cultivated. The liquid medium was separated from the mycelium by filtration. Medium fractions were extracted with ethyl acetate and isopropanol 9:1 volume:volume, and the mycelia with ethyl acetate and methanol 1:1. The extracts were vacuum dried and the larvicidal activity was evaluated in selective bioassays containing 500 μg/ml of the dried fungal extracts. Larval mortality was evaluated up to 72 h. None of the mycelium extracts showed larvicidal activity greater than 50% at 72 h. In contrast, 15 culture medium extracts had larvicidal activity equal to or greater than 50% and eight killed more than 90% of the larvae within 72 h. These eight extracts from fungi belonging to seven different genera (*Aspergillus, Cladosporium, Trichoderma, Diaporthe, Albifimbria, Emmia*, and *Sarocladium*) were selected for the determination of LC_50_ and LC_90_. *Albifimbria lateralis* (1160) medium extracts presented the lowest LC_50_ value (0.268 μg/ml) after 24 h exposure. *Diaporthe ueckerae* (1203) medium extracts presented the lowest value of LC_90_ (2.928 μg/ml) at 24 h, the lowest values of LC_50_ (0.108 μg/ml) and LC_90_ (0.894 μg/ml) at 48 h and also at 72 h (LC_50_ = 0.062 μg/ml and LC_90_ = 0.476 μg/ml). Extracts from *Al. lateralis* (1160) and *D. ueckerae* (1203) showed potential for developing new, naturally derived products, to be applied in integrated vector management programs against *Ae. aegypti*.

## Introduction

*Aedes aegypti* mosquitoes are the main vectors of arboviruses such as those that cause dengue, chikungunya, and Zika illnesses (Consoli and Oliveira, 1994; de Oliveira Barbosa Bitencourt et al., 2021). These diseases have occupied a prominent position in public health in several countries of the Americas, including Brazil where the occurrence of all these arboviruses has been recorded simultaneously since 2015 (Saúde and Saúde, 2016).

In 2014, chikungunya fever was first recorded in Brazil and spread rapidly throughout the country (Araújo et al., 2020). A short time later, Zika virus was detected in northeastern Brazil in 2015 (Possas et al., 2017). In 2016, the country reached a peak of cases, with more than 215 thousand estimated cases of Zika (Saúde and Saúde, 2018), which resulted in thousands of cases of neonatal microcephaly (Zanotto and Leite, 2018). Dengue is characterized as one of the main arboviruses with worldwide outbreaks occurring in the Americas, Africa, the Middle East, Asia, and the Pacific Islands. About 3.9 billion people in 129 countries are at risk of infection by the dengue virus, a notable increase from previous decades, partially explained by the improvement of records and recognition of the disease burden by governments (WHO, 2020). According to the Pan American Health Organization, the highest number of dengue cases ever reported globally was in 2019. Brazil alone reported about 2.2 million cases in 2019, representing 70% of the total recorded in the Americas (PAHO, 2020).

Since specific antiviral drugs and effective vaccines against these arboviruses are not available, measures to curb the transmission of these diseases remain focused on vector control, mostly through the elimination of breeding sites and the use of chemical insecticides (Zara et al., 2016). However, the frequent use of chemical insecticides is toxic to the environment and has resulted in the selection of insecticide-resistant mosquito populations (Seetharaman et al., 2018; Araújo et al., 2020). It is therefore urgently necessary to explore new approaches to control these vectors.

Fungal secondary metabolites constitute a rich source of bioactive molecules (Daniel et al., 2017), potentially useful for mosquito control. More specifically, fungi isolated from aquatic habitats are a rich and unexplored source of new natural products. In order to adapt and survive in the aquatic environment, fungi accumulate unique bioactive secondary metabolites, not found in terrestrial environments (Bhakuni and Rawat, 2006; Imhoff, 2016).

The Amazon rainforest contains ∼ 25% of the world’s terrestrial biodiversity (Malhi et al., 2008), including microorganisms potentially useful for *A. aegypti* control programs. In this work, we explored the larvicidal potential of the fungi isolated from the aquatic environments of the Amazon region. Our results suggest the possibility of utilizing fungi-derived extracts and/or their metabolites as part of integrated vector management programs.

## Materials and Methods

### Production of the Fungal Extracts

#### Fungi Isolation and Identification

Thirty-six fungi were isolated from water samples collected in the municipalities of Coari (muddy water) and São Gabriel da Cachoeira (black water), in the state of Amazonas, Brazil, using standard microbiological techniques. Water samples were collected at the following four sites: (a) Coari/C1 – dam (4° 06′ 43.7″ S 63° 07′ 43.6″ W), (b) Coari/C2 – natural lake (4° 06′ 56.6″ S 63° 08′ 34.4″ W), (c) São Gabriel da Cachoeira/S3 – fish farm (0° 6′ 54.873″ S 67° 5′ 12.859″ W), and (d) São Gabriel da Cachoeira/S4 – natural lake (0° 7′ 6.866″ S 67° 4′ 24.576″ W). Isolated fungi were preserved in glycerol 20%, at −80°C and stored in the collection of microorganisms of the Laboratory of Bioassays and Microorganisms of the Amazon at the Federal University of Amazonas (LabMicrA/UFAM). All fungi were registered in the Brazilian National System of Genetic Heritage Management and Associated Traditional Knowledge (SisGen) under the number AD64E07. The fungal strains were identified according to their unique deposit code in the LabMicrA/UFAM collection. Taxonomic identification of the strains was carried out in a previous study (Oliveira, 2021) and was based on the DNA sequences of the internal transcribed spacer region (ITS2) and macro- and micro morphological characters (Hanlin and Ulloa, 1988; Hawksworth et al., 1995; Dugan, 2006).

#### Fungal Extract Preparation

Each isolate was first inoculated in Petri dishes containing a PDA + L semi-solid culture medium (200 g/l potato, 20 g/l dextrose and 15 g/l agar and 2 g/l yeast extract). Three fragments of the mycelium of the fungi (three-point inoculation) were sown at equidistant points and cultivated at 26°C for 8 days to confirm the purity of the preserved samples. Then a single fragment of each fungus was transferred into a new Petri dish (central point) containing the PDA + L medium and grown under the same conditions used previously. Then, five fragments of 1 cm^2^ of each fungus were inoculated in 300 ml of PD + L liquid culture medium (200 g/l potato, 20 g/l dextrose, and 2 g/l yeast extract) under sterile conditions (Souza et al., 2004). The samples were prepared in quintuplicate, including the media control and kept in static mode at 26°C in the absence of light.

Glucose and pH measurements of all samples were carried out every 3 days using test strips (Uriclin 10). The optimal time of cultivation of each strain was established as the time needed for total consumption of the glucose provided in the fresh medium. The cultured liquid medium was then vacuum filtered and separated from the mycelium. The culture liquid, totalizing a final volume of 1.1 l for each fungus, was partitioned, so an organic mixture, immiscible with water, was required. The partitioning process was done in a separating funnel with a mixture of ethyl acetate (AcOEt) and isopropanol (iPr-OH) 9:1 volume/volume (v/v) three times, using each time 300 ml of the solvent mixture.

The mycelium extraction was an immersion process. The solvent mixture used polar and non-polarized directed metabolites. The mycelium fraction was soaked with a mixture of methanol (MeOH) and AcOEt 1:1 (v/v) for 48 h and was then filtered to obtain the first extract. The mycelium was soaked twice more for 24 h and the extracts were combined with the first one. Each liquid and mycelial extracts obtained were concentrated in a rotary evaporator (Tecnal^®^), under reduced pressure with a vacuum pump and at 45°C. Dried extracts were weighed and stored in a desiccator with activated silica.

### Rearing *Aedes aegypti*

Field collected *Aedes aegypti* eggs (F0) (Manaus, Brazil, February 2018) were placed in containers with water for hatching. The larvae were reared in a plastic tray containing distilled water, and the water was changed every 2 days. The larvae were fed daily with a mixture of rat food (Teklad Global 18%) and cat food (Whiskas^®^) at a ratio of 1:1 until they reached the pupal stage and were then transferred to plastic cups containing 50 ml of water, which were placed in mosquito rearing cages (30 cm × 30 cm × 30 cm) for the emergence of adult mosquitoes. *Aedes aegypti* taxonomic identification was confirmed by morphological examination of the emerging adults (Forattini, 2002).

Adults were fed with 10% sucrose solution soaked in cotton balls, and twice a week, the females were fed with blood by placing anesthetized hamsters (*Mesocricetus auratus*) on top of the entomological cage for 30 min, according to the protocol authorized by the Ethics Committee for the Use of Animals – CEUA (CEUA, opinion No. 054/2018). Plastic cups with 100 ml of water with partially immersed strip of filter paper were available for egg laying. The paper strips with laid eggs (F1) were dried for 2–3 days then placed in distilled water for hatching. The hatched larvae were again maintained in the same way as described before. Third instar larvae of the second generation (F2) were used for the larvicidal bioassays. All mosquitoes were kept under controlled conditions of temperature of 26 ± 2°C and relative humidity of 75 ± 5%, with a photoperiod of 12:12 h (light/dark), as recommended by the WHO (2005).

### Larvicidal Bioassays

The selective and quantitative bioassays followed the criteria established by Dulmage et al. (1990) and the WHO (2005) with minor modifications. All bioassays were conducted under temperature, humidity, and photoperiod-controlled conditions, as previously mentioned.

Selective bioassays were performed in triplicate using 50 ml plastic cups containing 10 ml of distilled water, ten 3rd instar larvae, powdered rat food (Teklad Global 18%) and 500 μg/ml of the fungal extract. All tested samples were solubilized in dimethyl sulfoxide (DMSO; Thermo Fisher Scientific). Mortality readings were recorded at 24, 48, and 72 h after exposure to the fungal extracts (Danga et al., 2014). The extracts that presented mortality equal to or greater than 90% in the selective bioassay were chosen to perform quantitative bioassays and determine lethal concentrations able to kill 50% (LC_50_) or 90% of the larvae (LC_90_).

To determine LC_50_ and LC_90_ values, larvae were exposed to eight different concentrations of the fungal extracts, ranging from 0.01 to 250 μg/ml. Each concentration was tested in quintuplicate with three repetitions. All assays were conducted in plastic cups with a capacity of 110 ml, containing 20 ml of distilled water, powdered food, twenty 3rd instar larvae and the quantity corresponding to each concentration of fungal extract tested. DMSO as the negative control and Temephos (Pestanal Sigma-Aldrich) as the positive control were used at the same concentrations as the extracts. DMSO (maximum volume of DMSO in the assay – 0.1 ml) did not cause mortality in any of the tested concentrations and Temephos (500 μg/ml) killed 100% of the larvae in the selective bioassay.

### Statistical Analysis

The mortality data obtained in the bioassays were submitted to Probit analysis *p* ≤ 0.05 (Finney, 1952), using the statistical software Polo Plus (LeOra Software, CA, United States; Haddad, 1998). Lethal concentrations and the confidence interval (95% CI) were calculated using the Lilliefors normality test (K), analysis of variance (ANOVA), a multiple comparison test (*p* ≤ 0.05) and the Student’s *t* test using BioStat 5.3 for Windows software (Ayres et al., 2007).

## Results

In this study, 36 isolates belonging to 23 genera of fungi were analyzed regarding their ability to produce mosquito larvicidal compounds. Extracts from isolated strain were obtained from both mycelium and culture liquid medium fractions of the cultures. The growth time of the fungi until no glucose was detected in the medium ranged from 17 to 85 days. The pH of the cultures ranged from 6 to 7.5 in comparison to the pH of 5.5 in the control (non-inoculated medium). The extracts obtained from the liquid medium presented yielded between 82 and 724 mg, after extraction and drying procedures. Mycelium extracts from fungi strains 1132 and 1126 yielded 256 and 5872 mg, respectively, being the lowest and the highest obtained values (Table 1).

**TABLE 1:** Crude extracts of 36 fungi isolated from aquatic environments in the Amazonian municipalities of Coari and São Gabriel da Cachoeira used in the larvicidal tests against *Aedes aegypti*.

Tested lineage	GenBank accession numbers	Taxonomic identification	Cultivation time (days)	pH	Glucose	Extract yield (mg)	
						Liquid medium	Mycelium
1160	MZ781268	*Albifimbria lateralis* ^*C*1^	50	6.5	0	120	820
1283	MZ781299	*Aspergillus hortai* ^*C*1^	17	6.5	0	724	2577
1126	MZ781261	*Aspergillus* sp. ^*C*1^	24	6.5	0	254	5872
1169	MZ781272	*Chrysoporthe* sp. ^*C*1^	18	6.5	0	260	1624
1132	MZ781262	*Cladosporium* sp. ^*C*1^	17	7	0	134	256
1135	MZ781264	*Cladosporium* sp. ^*C*1^	17	6.5	0	177	334
1098	MZ781256	*Cytospora* sp. ^*C*2^	52	6	0	180	628
1106	MZ781257	*Cytospora* sp. ^*C*2^	41	6	0	443	3123
1203	MZ781276	*Diaporthe ueckerae* ^*S*4^	41	6	0	249	2298
1242	MZ781281	*Diaporthe ueckerae ^*S*4^*	28	7	0	152	872
1232	MZ781279	*Emmia* sp. ^*S*4^	52	7.5	0	161	1128
1248	MZ781286	*Epicoccum latusicollum* ^*C*1^	24	6.5	0	134	2564
1240	MZ781280	*Eutypella* sp. ^*S*4^	41	6.5	0	221	3393
1262	MZ781291	*Fusarium oxysporum* ^*C*1^	24	6.5	0	82	1559
1280	MZ781298	*Fusarium oxysporum* ^*C*1^	24	6.5	0	98	959
1085	MZ781250	*Fusarium* sp. ^*S*4^	27	7	0	90	1430
1277	MZ781297	*Hongkongmyces* sp. ^*S*4^	72	6.5	0	92	439
1273	MZ781296	*Hyphodermella* sp. ^*C*1^	67	6	0	85	547
1205	MZ781277	*Hypomontagnella monticulosa* ^*C*1^	72	8	0	258	750
1082	MZ781248	*Microsphaeropsis arundinis^ C1^*	52	7.5	0	129	947
1079	MZ781246	*Nigrograna chromolaenae* ^*C*1^	55	6.5	0	117	2840
1123	MZ781259	Ochronis sp. ^*C*1^	28	6.5	0	149	540
1083	MZ781249	*Paraconiothyrium estuarinum* ^*C*1^	28	6.5	0	129	2830
1184	MZ781274	*Paraconiothyrium estuarinum ^*S*4^*	63	6.5	0	140	2019
1265	MZ781293	*Paraconiothyrium estuarinum* ^*C*1^	27	7	0	138	1850
1080	MZ781247	*Paraconiothyrium* sp. ^*S*4^	28	6	0	132	1985
1245	MZ781283	*Penicillium citreosulfuratum* *^S^* ^4^	35	6	0	250	838
1266	MZ781294	*Sorocladium* sp. ^*C*2^	80	6.5	0	92	645
1089	MZ781252	*Striaticonidium synnematum* ^*S*3^	31	7.5	0	205	1025
1263	MZ781292	*Talaromyces amestolkiae* ^*C*2^	18	6	0	250	3028
1087	MZ781251	*Talaromyces* sp. ^*C*2^	50	6.5	0	268	820
1244	MZ781282	*Talaromyces* sp. ^*S*4^	80	6.5	0	262	901
1246	MZ781284	*Talaromyces* sp. ^*S*4^	17	6	0	127	1292
1247	MZ781285	*Trametes menziesii* ^*C*2^	35	6	0	158	2870
1133	MZ781263	*Trichoderma atroviride* ^*C*2^	80	7.5	0	134	2109
1136	MZ781265	*Trichoderma atroviride* ^*C*2^	85	6	0	223	334
		Control (culture medium)	80	5.5	2000	201	–

*The water samples were collected at the following four sites: (a) ^C1^ Coari – dam; (b) ^C2^ Coari – natural lake; (c) ^S3^ São Gabriel da Cachoeira – fish rearing pond; and (d) ^S4^ São Gabriel da Cachoeira – natural lake. GenBank accession numbers are nucleotide sequences of approximately 700 bp including the internal transcribed spacers (ITS1-5.8S-ITS2). Cultivation time is the time needed for total consumption of the glucose provided in the fresh medium. Extract yield is the dry weight of extracted metabolites.*

Seven mycelium extracts originating from fungi belonging to six genera (*Aspergillus, Cladosporium, Fusarium, Diaporthe, Talaromyces*, and *Trichoderma*) caused larval mortality from 3.3 to 43.3%, and none presented mortality equal to or greater than 50% up to 72 h of exposure (Supplementary Table 1). Larvicidal activity equal to or greater than 50% was observed in 15 of the 36 extracts of liquid medium; six liquid medium extracts belonging to five genera (*Albifimbria, Aspergillus, Diaporthe, Emmia*, and *Sorocladium*) killed 100% of the larvae within 72 h.

**FIGURE 1:**
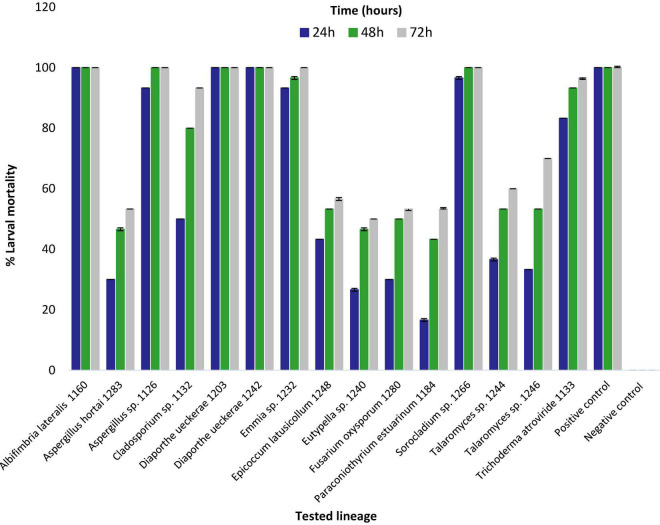
Percentage of mortality of 3rd instar *Aedes aegypti* larvae exposed to liquid medium extracts obtained from strains of fungi isolated from Amazonian aquatic environments. Mortality was assessed after 24, 48, and 72 h of exposure to fungal extract at a concentration of 500 μg/ml. Error bars represent standard deviation. Positive control (Temephos) and Negative control (dimethyl sulfoxide).

Eight extracts (from strains 1126, 1132, 1133, 1160, 1203, 1232, 1242, and 1266) showed larvicidal activity equal to or greater than 50% at 24 h, four (1244, 1246, 1248, and 1280) resulted in 50% mortality only at 48 h and three extracts (1184, 1240, and 1283) caused 50% mortality only at 72 h of exposure. Six extracts caused 100% larval mortality, three (1160, 1203, and 1242) in less than 24 h, two (1126 and 1266) at 48 h and one (1232) at 72 h (Figure 1).

Extracts with larvicidal activity equal to or greater than 90% (*Albifimbria lateralis* 1160, *Aspergillus* sp. 1126, *Cladosporium* sp. 1132, *D. ueckerae* 1203 and 1242, *Emmia* sp. 1232, *Sorocladium* sp. 1266, and *Trichoderma atroviride* 1133) were further studied and LC_50_ and LC_90_ values determined (Figure 1 and Supplementary Table 1).

Overall, the liquid medium extracts from *Al. lateralis* 1160 and *D. ueckerae* 1203 showed the best results, with highest mortality rates and lowest LC values. The extract of the strain 1160 (*Al. lateralis*) presented the lowest LC_50_ (0.268 μg/ml) at 24 h. The extract of the 1203 strain (*D. ueckerae*) had the lowest LC_90_ (2.928 μg/ml) at 24 h. Furthermore, *D. ueckerae* 1203 extracts had the lowest LC_50_ (0.108 μg/ml) and LC_90_ (0.894 μg/ml) at 48 h, and at 72 h with an LC_50_ of 0.062 μg/ml and an LC_90_ of 0.476 μg/ml (Table 2).

**TABLE 2:** Lethal larvicidal concentration of liquid culture medium extracts obtained from fungal strains against 3rd instar *Aedes aegypti* larvae.

Tested lineage	LC_50_ μg/ml (CI 95%)	LC_90_ μg/ml (CI 95%)	χ^2^	Df	Slope ± SE
**24 h**					
Tp	0.025 (0.003–0.063)ae	1.161 (0.647–3.364)a	6.9432	5	0.768 ± 0.075
1126	0.872 (0.200–3.956)abd	67.251 (9.676–0.228E + 06)ac	2.8009	2	0.679 ± 0.066
1132	0.459 (0.043–1.057)abd	74.117 (11.419–0.152E + 07)ac	3.9608	3	0.580 ± 0.057
1133	0.463 (0.367–0.586)bh	10.940 (6.789–20.553)bc	1.000	6	0.933 ± 0.060
1160	*0.268 (0.211–0.332)cdf	3.384 (2.429–5.223)ac	2.995	5	1.164 ± 0.061
1203	0.461 (0.123–0.789)adb	*2.928 (1.565–12.543)ac	9.5913	3	1.597 ± 0.065
1232	0.372 (0.087–0.731)adh	26.304 (6.548–35.519)ac	7.6254	4	0.693 ± 0.054
1242	0.427 (0.029–0.839)ah	67.918 (15.092–68.770)ac	0.460	2	0.582 ± 0.078
1266	1.904 (1.288–3.317)ef	205.87 (53.346–272.74)ac	0.961	4	0.630 ± 0.053
**48 h**					
Tp	0.016 (0.002–0.038)a	0.322 (0.203–0.555)a	5.3850	5	0.979 ± 0.111
1126	0.337 (0.191–0.489)ab	6.293 (3.826–14.549)a	1.922	2	1.009 ± 0.069
1132	0.170 (0.026–0.351)ab	9.896 (3.537–20.621)a	6.3742	4	0.726 ± 0.057
1133	0.142 (0.050–0.253)ab	3.261 (1.548–15.627)a	12.854	5	0.942 ± 0.063
1160	0.123 (0.058–0.195)b	1.106 (0.666–2.735)a	13.497	5	1.345 ± 0.083
1203	*0.108 (0.062–0.157)b	*0.894 (0.604–1.637)a	8.1443	5	1.397 ± 0.091
1232	0.206 (0.030–0.412)b	2.876 (1.302412–35.975)a	15.328	4	1.119 ± 0.074
1242	0.140 (0.078–0.211)b	12.095 (5.974–37.560)a	1.821	5	0.662 ± 0.056
1266	0.391 (0.295–0.505)c	10.825 (6.378–22.952)a	3.846	5	0.889 ± 0.091
**72 h**					
Tp	0.025 (0.009–0.041)a	0.141 (0.107–0.185)a	2.532	5	1.694 ± 0.280
1126	0.120 (0.053–0.198)a	5.877 (3.351–15.174)a	1.393	4	0.759 ± 0.059
1132	0.079 (0.033–0.132)a	1.387 (0.847–3.138)a	7.5183	5	1.028 ± 0.074
1133	0.070 (0.023–0.126)a	0.890 (0.531–2.237)a	11.466	5	1.163 ± 0.086
1160	0.088 (0.055–0.122)a	0.692 (0.506–1.074)a	5.2154	5	1.434 ± 0.101
1203	*0.062 (0.024–0.103)a	*0.476 (0.315–0.941)a	10.131	5	1.453 ± 0.121
1232	0.096 (0.031–0.172)a	1.292 (0.713–4.132)a	14.264	5	1.134 ± 0.077
1242	0.088 (0.030–0.159)a	2.119 (1.139–6.806)a	9.3874	5	0.928 ± 0.068
1266	0.180 (0.101–0.269)a	2.216 (1.307–5.364)a	9.8043	5	1.174 ± 0.101

The LC_50_ and LC_90_ were determined at 24, 48, and 72 h after exposure to fungal extracts. LC, lethal concentration; CI, confidence interval; χ^2^-chi, square; Df, degrees of freedom; SE, standard error. Equal letters (a, b, c, etc.) do not differ in the probability level of 5% (p > 0.05), Tp, Temephos (positive control). The lowest LC values for each time evaluated are shaded in gray and marked with *.

## Discussion

The public health importance of *Ae. aegypti* in tropical regions has attracted the attention of local authorities and the World Health Organization due to the wide geographical distribution and severity of diseases transmitted by these mosquitoes in the last decades, especially dengue, chikungunya, and Zika (Rodrigues-Alves et al., 2020). As such, there is a growing interest in new insecticides and larvicides capable of controlling this vector. Naturally derived insecticides have been pursued as potentially less toxic alternatives, aiming at reducing environmental pollution and preventing the selection of mosquitoes resistant to chemical insecticides (Al-Mekhlafi, 2017; Araújo et al., 2020).

For the control of *Ae. aegypti*, insecticides are frequently applied directly in natural bodies of water and/or artificial containers, usually located closely or kept inside human households. Therefore, the use of natural, potentially less-toxic, insecticides is desirable from both environmental and social perspectives. This work investigated for the first time extracts of fungi isolated from aquatic habitats of the Amazon region in order to identify fungal lineages that can produce larvicidal bioactive metabolites against *Ae. aegypti*.

Following the protocols described here, none of the mycelium extracts showed larvicidal activity resulting in mortality rates above 50% within 72 h. However, 15 extracts from the liquid culture medium resulted in more than 50% mortality. Six killed 100% of the larvae within 72 h and three of these were lethal in less than 24 h, resembling the positive control Temephos. These results demonstrate that some of the isolated fungal strains secrete metabolites with larvicidal activity against *Ae. aegypti*.

The fungi tested in this study were subjected to the similar cultivation conditions. However, cultivation times varied for each fungal strain. To avoid differences in nutrient availability, we used total glucose consumption in the culture medium as determinant of the cultivation time length. Fungi growth styles and physical, chemical and biological factors, among others, influence development time and the production of bioactive metabolites (Kavanagh, 2011; Costa and Nahas, 2012). Species-specific traits explain the difference in cultivation time and biological activities among the isolated fungi studied in our work.

Mosquito larvicidal activities of the mycelial extracts and the liquid culture medium extracts, have been described for other fungi such as *Stereum* sp. (JO5289) (Chirchir et al., 2013), *Beauveria bassiana* (UNI 40) (Daniel et al., 2017), *Trametes* sp. (Waweru et al., 2017), *Pestalotiopsis virgulata* and *Pycnoporus sanguineus* (Bücker et al., 2013). However, the LC_50_ values revealed in our work are lower than those previously published, such as *Metarhizium anisopliae* (LC_50_ = 59.83 μg/ml, Vivekanandhan et al., 2020), *B. bassiana* (LC_50_ = 1.230 μg/ml, Daniel et al., 2017), and *Aspergillus terreus* (LC_50_ = 80.407 μg/ml, Ragavendran and Natarajan, 2015), indicating the potency of the metabolites obtained from the strains tested in our study.

It is worth noting that to date there have been no reports of biological activities against insect species of metabolites produced by fungal strains of the species *Al. lateralis, D. ueckerae*, and *Emmia* sp. For the first time, lineages of these species of fungi with larvicidal activity against *Ae. aegypti* have been identified.

The genus *Albifimbria* consists of four species, i.e., *Al. lateralis*, *Albifimbria terrestris, Albifimbria verrucaria*, and *Albifimbria viridis*, which are usually found in soil, leaves, fruits, and in the air (Lombard et al., 2016). The species *Albifimbria lateralis* (Lombard et al., 2016) has been recently described and needs better investigation regarding the production of secondary metabolites, though our investigation indicates promising applications of this fungus species in vector control. Metabolites produced by *Al. verrucaria* exhibit antimicrobial activities (Zou et al., 2011) and bioherbicidal activities (Walker and Tilley, 1997) and are considered to be a potential biocontrol agent against the fungus *Botrytis cinerea* in grapes (Li et al., 2019).

The species *D. ueckerae* was described by Udayanga et al. (2015). Its occurrence in Brazil was identified by Soares et al. (2018) who isolated this species of fungus from *Costus spiralis* (Jacq.) Roscoe (Costaceae), a plant native to the Amazon region used in traditional medicine. Fungal species of the genus *Diaporthe* are known to be a rich source of secondary metabolites (Chepkirui and Stadler, 2017).

Currently, 106 compounds derived from *Diaporthe* exhibiting biological activities, such as cytotoxic, antifungal, antibacterial, antiviral, antioxidant, anti-inflammatory, phytotoxic, antiparasitic, and herbicidic activities, have been studied (Ash et al., 2010; Meepagala et al., 2018; Xu et al., 2021). Two cyclohexeneoxidediones, phyllostine acetate (1) and phyllostine (2), from the fungus *Diaporthe miriciae*, showed insecticidal activity against *Plutella xylostella* larvae (Ratnaweera et al., 2020). Meepagala et al. (2018) isolated a compound from the liquid medium extract of *Diaporthe eres*, identified as 3,4-dihydro-8-hydroxy-3,5-dimethylisocoumarin (1), which has larvicidal activity against *Ae. aegypti*.

In addition, other fungi from different genera such as *Beauveria*, *Fusarium*, *Metarhizium*, *Neosartorya*, and *Paecilomyces*, also produce compounds with insecticidal activity such as beauvericin, gliotoxin, enniatin, oosporein, destruxins, cytochalasins, etc (Vyas et al., 2007; Masi et al., 2017; Berestetskiy and Hu, 2021).

## Conclusion

This study is the first to evaluate aquatic fungi strains from the Amazon for their ability of producing mosquito larvicidal metabolites. Our findings open opportunities for the development of new larvicides that may be used as mosquito control agents. Crude fungal extracts, such as those studied here, are a complex mixture of different classes of molecules. The process of fractionation and purification of raw extracts guided by bioactivity (Chirchir et al., 2013) is necessary for the isolation and characterization of the chemical compounds responsible for the larvicidal activities observed in our work. Further studies are needed to characterize the active larvicidal metabolites produced by these fungi and define their mechanisms of action.

## Data Availability Statement

The original contributions presented in the study are included in the article/Supplementary Material, further inquiries can be directed to the corresponding authors.

## Author Contributions

MO, WT, ADS, and AQS designed the study. MO, ADS, and AQS performed the production of fungal extracts, analyzed the results, and wrote the manuscript. MO and RK reared mosquitoes and carried out bioassays. MO, GS, OM, OT, ADS, and AQS supervised and finalized the manuscript. All authors read and approved the final manuscript.

## Conflict of Interest

The authors declare that the research was conducted in the absence of any commercial or financial relationships that could be construed as a potential conflict of interest.

## Publisher’s Note

All claims expressed in this article are solely those of the authors and do not necessarily represent those of their affiliated organizations, or those of the publisher, the editors and the reviewers. Any product that may be evaluated in this article, or claim that may be made by its manufacturer, is not guaranteed or endorsed by the publisher.
