# Supplementary material for: Extracts of Amazonian Fungi With Larvicidal Activities Against Aedes aegypti
Source: Front Microbiol. 2021 Dec 10;12:743246. doi: 10.3389/fmicb.2021.743246 (PMC8702858; doi:10.3389/fmicb.2021.743246)
Supplement: Supplementary file 1 [file Table_1.docx]

Supplementary Material

# Supplementary Table

| **Taxonomic identification** | **Tested strain** | **% of larval mortality ± SD** | | | | | | |
| --- | --- | --- | --- | --- | --- | --- | --- | --- |
|  |  | **Liquid medium extract** | | |  | **Mycelium extract** | | |
|  |  | 24 h | 48 h | 72 h |  | 24 h | 48 h | 72 h |
| *Albifimbria lateralis* | 1160 | 100  ± 00 | 100  ± 00 | 100  ± 00 |  | 0  ± 00 | 0  ± 00 | 0  ± 00 |
| *Aspegillus hortai* | 1283 | 30  ± 00 | 46,6  ± 0,47 | 53,3  ± 0,47 |  | 0  ± 00 | 0  ± 00 | 0  ± 00 |
| *Aspergillus* sp. | 1126 | 93,3  ± 0,47 | 100  ± 00 | 100  ± 00 |  | 16,6  ± 0,47 | 23,3  ± 00 | 23,3  ± 00 |
| *Chrysoporthe* sp. | 1169 | 16,6  ± 0,47 | 16,6  ± 0,47 | 16,6  ± 0,47 |  | 0  ± 00 | 0  ± 00 | 0  ± 00 |
| *Cladosporium* sp. | 1132 | 50  ± 00 | 80  ± 00 | 93,3  ± 0,47 |  | 10  ± 00 | 26,6  ± 0,47 | 33,3  ± 00 |
| *Cladosporium* sp. | 1135 | 3,3  ± 0,47 | 26,6  ± 0,47 | 43,3  ± 0,47 |  | 0  ± 00 | 0  ± 00 | 0  ± 00 |
| *Cytospora* sp. | 1098 | 3,3  ± 0,47 | 3,3  ± 0,47 | 3,3  ± 0,47 |  | 0  ± 00 | 0  ± 00 | 0  ± 00 |
| *Cytospora* sp. | 1106 | 13,3  ± 0,47 | 13,3  ± 0,47 | 13,3  ± 0,47 |  | 0  ± 00 | 0  ± 00 | 0  ± 00 |
| *Diaporthe ueckerae* | 1203 | 100  ± 00 | 100  ± 00 | 100  ± 00 |  | 6,6  ± 0,47 | 10  ± 00 | 10  ± 00 |
| *D. ueckerae* | 1242 | 100  ± 00 | 100  ± 00 | 100  ± 00 |  | 0  ± 00 | 0  ± 00 | 0  ± 00 |
| *Emmia* sp. | 1232 | 93,3  ± 0,47 | 96,6  ± 0,47 | 100  ± 00 |  | 0  ± 00 | 0  ± 00 | 0  ± 00 |
| *Epicoccum latusicollum* | 1248 | 43,3  ± 0,94 | 53,3  ± 0,47 | 56,6  ± 1,24 |  | 0  ± 00 | 0  ± 00 | 0  ± 00 |
| *Eutypella* sp. | 1240 | 26,6  ± 0,47 | 46,6  ± 0,47 | 50  ± 00 |  | 0  ± 00 | 0  ± 00 | 0  ± 00 |
| *Fusarium oxysporum* | 1262 | 0  ± 00 | 6,6  ± 0,94 | 16,6  ± 1,6 |  | 0  ± 00 | 4  ± 0,47 | 6  ± 0,47 |
| *F. oxysporum* | 1280 | 30  ± 00 | 50  ± 00 | 53,3  ± 0,47 |  | 0  ± 00 | 0  ± 00 | 0  ± 00 |
| *Fusarium* sp. | 1085 | 16,6  ± 0,94 | 23,3  ± 0,62 | 30  ± 0,81 |  | 0  ± 00 | 3,3  ± 0,47 | 3,3  ± 0,47 |
| *Hongkongmyces* sp. | 1277 | 33,3  ± 0,47 | 46,6  ± 1,24 | 46,6  ± 1,24 |  | 0  ± 00 | 0  ± 00 | 0  ± 00 |
| *Hyphodermella* sp*.* | 1273 | 3,3  ± 0,47 | 6,6  ± 0,47 | 6,6  ± 0,47 |  | 0  ± 00 | 0  ± 00 | 0  ± 00 |
| *Hy. monticulosa* | 1205 | 23,3  ± 0,47 | 23,3  ± 0,47 | 23,3  ± 0,47 |  | 0  ± 00 | 0  ± 00 | 0  ± 00 |
| *Microsphaeropsis arundinis* | 1082 | 0  ± 00 | 0  ± 00 | 0  ± 00 |  | 0  ± 00 | 0  ± 00 | 0  ± 00 |
| *Nigrograna chromolaenae* | 1079 | 0  ± 00 | 0  ± 00 | 0  ± 00 |  | 0  ± 00 | 0  ± 00 | 0  ± 00 |
| *Ochronis* sp. | 1123 | 3,3  ± 0,47 | 3,3  ± 0,47 | 3,3  ± 0,47 |  | 0  ± 00 | 0  ± 00 | 0  ± 00 |
| *Paraconiothyrium estuarinum* | 1083 | 6,6  ± 0,47 | 10  ± 1,4 | 13,3  ± 1,24 |  | 0  ± 00 | 0  ± 00 | 0  ± 00 |
| *P. estuarinum* | 1184 | 16,6  ± 0,47 | 43,3  ± 0,94 | 53,3  ± 0,47 |  | 0  ± 00 | 0  ± 00 | 0  ± 00 |
| *P. estuarinum* | 1265 | 16,6  ± 0,94 | 26,6  ± 1,24 | 43,30  ± 1,24 |  | 0  ± 00 | 0  ± 00 | 0  ± 00 |
| *Paraconiothyrium* sp. | 1080 | 13,3  ± 0,47 | 20  ± 00 | 20  ± 00 |  | 0  ± 00 | 0  ± 00 | 0  ± 00 |
| *Penicillium citreosulfuratum* | 1245 | 23,3  ± 0,47 | 26,6  ± 0,47 | 33,3  ± 0,47 |  | 0  ± 00 | 0  ± 00 | 0  ± 00 |
| *Sarocladium* sp. | 1266 | 96,6  ± 0,47 | 100  ± 00 | 100  ± 00 |  | 0  ± 00 | 0  ± 00 | 0  ± 00 |
| *Striaticonidium synnematum* | 1089 | 0  ± 00 | 0  ± 00 | 0  ± 00 |  | 0  ± 00 | 0  ± 00 | 0  ± 00 |
| *Talaromyces amestolkiae* | 1263 | 0  ± 00 | 0  ± 00 | 3,3  ± 0,47 |  | 0  ± 00 | 23,3  ± 0,47 | 40  ± 00 |
| *Talaromyces* sp. | 1087 | 0  ± 00 | 3,3  ± 0,47 | 6,6  ± 0,47 |  | 0  ± 00 | 0  ± 00 | 0  ± 00 |
| *Talaromyces* sp. | 1244 | 36,6  ± 0,47 | 53,3  ± 0,47 | 60  ± 00 |  | 0  ± 00 | 0  ± 00 | 0  ± 00 |
| *Talaromyces* sp. | 1246 | 33,3  ± 0,47 | 53,3  ± 0,47 | 70  ± 0,81 |  | 0  ± 00 | 0  ± 00 | 0  ± 00 |
| *Trametes menziesii* | 1247 | 0  ± 00 | 0  ± 00 | 0  ± 00 |  | 0  ± 00 | 0  ± 00 | 0  ± 00 |
| *Trichoderma atroviride* | 1133 | 83,3  ± 0,47 | 93,3  ± 0,47 | 96,6  ± 0,47 |  | 0  ± 00 | 0  ± 00 | 0  ± 00 |
| *T. atroviride* | 1136 | 6,6  ± 0,47 | 16,6  ± 0,47 | 26,6  ± 0,47 |  | 13,3  ± 0,47 | 40  ± 00 | 43,3  ± 0,81 |
| Positive control (Temephos) | | 100  ± 00 | 100  ± 00 | 100  ± 00 |  | 100  ± 00 | 100  ± 00 | 100  ± 00 |
| Negative control (DMSO) | | 0  ± 00 | 0  ± 00 | 0  ± 00 |  | 0  ± 00 | 0  ± 00 | 0  ± 00 |

**Supplementary Table 1**. Larvicidal activity of extracts obtained from the liquid culture medium and from the mycelium of fungi isolated from aquatic environments in the Amazonian municipalities of Coari and São Gabriel da Cachoeira against 3^rd^ instar larvae of *Aedes aegypti.* Mortality was assessed at 24, 48 and 72 h after exposure to the fungal extract at a concentration of 500 µg/mL. The extracts that presented 90% mortality in up to 72 h are shaded in gray. Standard Deviation (SD) and dimethylsulfoxide (DMSO).
